# Supplementary material for: Flavinated SDHA underlies the change in intrinsic optical properties of oral cancers
Source: Commun Biol. 2023 Nov 9;6:1134. doi: 10.1038/s42003-023-05510-w (PMC10636189; doi:10.1038/s42003-023-05510-w)
Supplement: Supplementary file 2 — Description of Additional Supplementary Files [file 42003_2023_5510_MOESM2_ESM.pdf]

Description of Additional Supplementary Files

File name: Supplementary Data
Description: Numerical source data for graphs and charts corresponding to Figures 3a, 3b, 3c, 4b, 5b, 6a, 6b, 6c, 6d, 6e, 6f and Supplementary Figures 1a, 1b.
