# Supplementary material for: Flavinated SDHA underlies the change in intrinsic optical properties of oral cancers
Source: Commun Biol. 2023 Nov 9;6:1134. doi: 10.1038/s42003-023-05510-w (PMC10636189; doi:10.1038/s42003-023-05510-w)
Supplement: Supplementary file 4 — Reporting Summary [file 42003_2023_5510_MOESM4_ESM.pdf]

nature portfolio

Corresponding author(s): Christopher H. Contag

Last updated by author(s): 10/19/2023

Reporting Summary

Nature Portfolio wishes to improve the reproducibility of the work that we publish. This form provides structure for consistency and transparency in reporting. For further information on Nature Portfolio policies, see our Editorial Policies and the Editorial Policy Checklist.

Statistics

For all statistical analyses, confirm that the following items are present in the figure legend, table legend, main text, or Methods section.

n/a	Confirmed	
☐	☒	The exact sample size (*n*) for each experimental group/condition, given as a discrete number and unit of measurement
☐	☒	A statement on whether measurements were taken from distinct samples or whether the same sample was measured repeatedly
☐	☒	The statistical test(s) used AND whether they are one- or two-sided<br>*Only common tests should be described solely by name; describe more complex techniques in the Methods section.*
☒	☐	A description of all covariates tested
☐	☒	A description of any assumptions or corrections, such as tests of normality and adjustment for multiple comparisons
☐	☒	A full description of the statistical parameters including central tendency (e.g. means) or other basic estimates (e.g. regression coefficient) AND variation (e.g. standard deviation) or associated estimates of uncertainty (e.g. confidence intervals)
☐	☒	For null hypothesis testing, the test statistic (e.g. *F*, *t*, *r*) with confidence intervals, effect sizes, degrees of freedom and *P* value noted<br>*Give P values as exact values whenever suitable.*
☒	☐	For Bayesian analysis, information on the choice of priors and Markov chain Monte Carlo settings
☒	☐	For hierarchical and complex designs, identification of the appropriate level for tests and full reporting of outcomes
☒	☐	Estimates of effect sizes (e.g. Cohen's *d*, Pearson's *r*), indicating how they were calculated

Our web collection on statistics for biologists contains articles on many of the points above.

Software and code

Policy information about availability of computer code

Data collection: Pathologist-based tumor regions were annotated on slides using Aperio's annotation software (ImageScope Viewing Software: Positive Pixel Count v9.1, Aperio ImageScope®, Leica Microsystems Inc.).

Data analysis: Data were analyzed using GraphPad Prism version 8.4.3 (GraphPad Software, San Diego CA; www.graphpad.com); Comparative analysis of proteins extracted from gels by LC/MS/MS were analyzed using Scaffold, version 4.8.9 (www.proteomesoftware.com). Western blot images were analyzed using ImageJ (version 1.53k).

For manuscripts utilizing custom algorithms or software that are central to the research but not yet described in published literature, software must be made available to editors and reviewers. We strongly encourage code deposition in a community repository (e.g. GitHub). See the Nature Portfolio guidelines for submitting code & software for further information.

Data

Policy information about availability of data

All manuscripts must include a data availability statement. This statement should provide the following information, where applicable:

- Accession codes, unique identifiers, or web links for publicly available datasets
- A description of any restrictions on data availability
- For clinical datasets or third party data, please ensure that the statement adheres to our policy

The data supporting the findings of this study are available within the paper and its Supplementary Information.

Human research participants

Policy information about studies involving human research participants and Sex and Gender in Research.

Reporting on sex and gender	Among subjects, 20% was female (and identified as women based on self-reporting) and 80% was male (and identified as men based on self-reporting). Neither sex nor gender was considered a variable in our study design, as the number of patients available for this study did not allow for robust statistical comparison of sex nor gender.
Population characteristics	Briefly, subjects' age ranged 32-78 years. All subjects were diagnosed with primary head and neck squamous cell carcinoma located on the lateral tongue (n = 2), the floor of the mouth (n = 1), in the larynx (n = 1) or on the alveolar ridge (n = 1). Subject's tumor stage was cT3 in 2 cases, cT4a in 2 cases, and one patient presented with recurrent larynx cancer that was previously treated with chemo-radiation (cT-stage unknown). Pathological cancer stage was pT3 in 1 subject, pT4a in two subjects and pT2 in 2 subjects. None of the subjects reported on alcohol abuse, and 2 out of 5 subjects never smoked, one subject quit smoking and two subjects reported to be current smokers. Three subjects were HPV negative, one positive and one unknown. Separately, the Illumiscan was used to examine loss of fluorescence intensity in an 86-year old female diagnosed with T1 N0 M0 tongue cancer on the right side.
Recruitment	Patients were recruited both from the head and neck oncology practice at Stanford and from Tokyo Dental College. At Stanford, all patients were evaluated by Dr. Rosenthal. Written Informed Consent and HIPAA Authorization were obtained after the Informed Consent was reviewed and the study was fully explained to each patient. The protocol adhered to regulations to provide protection for human subjects in clinical investigations described by the general requirements for informed consent. The DSMB of Stanford University provided oversight for the trial and trial safety.<br><br>Bias: To the best of our knowledge, self-selection bias did not occur.
Ethics oversight	This study protocol was approved by the Stanford University Institutional Review Board (IRB-35064) and the FDA (NCT02415881) with written informed consent obtained from all patients. Similarly, the study protocol at Tokyo Dental College was approved by the ethics committees of Tokyo Dental College (Approval number: 740), after obtaining patient consent. The study was performed in accordance with the Helsinki Declaration of 1975 and its amendments, FDA's ICH-GCP guidelines, and the laws and regulations of the United States.

Note that full information on the approval of the study protocol must also be provided in the manuscript.

Field-specific reporting

Please select the one below that is the best fit for your research. If you are not sure, read the appropriate sections before making your selection.

☒ Life sciences ☐ Behavioural & social sciences ☐ Ecological, evolutionary & environmental sciences

For a reference copy of the document with all sections, see nature.com/documents/nr-reporting-summary-flat.pdf

Life sciences study design

All studies must disclose on these points even when the disclosure is negative.

Sample size	Sample sizes depended on specific experiments and were determined by the magnitude and consistency of measurable differences between groups; and similar studies in the field. For human studies, sample size was based on the number of available patients included in our phase I study at the time.
Data exclusions	An outlier was statistically excluded using the Grubb's test as described in the legend for Fig. 3a.
Replication	Result from each in-vitro experiment was independently reproduced with similar results.
Randomization	No randomizations were performed.
Blinding	Blinding was not performed given the nature of the study.

Reporting for specific materials, systems and methods

We require information from authors about some types of materials, experimental systems and methods used in many studies. Here, indicate whether each material, system or method listed is relevant to your study. If you are not sure if a list item applies to your research, read the appropriate section before selecting a response.

Materials & experimental systems

n/a	Involved in the study	
☐	☒	Antibodies
☐	☒	Eukaryotic cell lines
☒	☐	Palaeontology and archaeology
☒	☐	Animals and other organisms
☐	☒	Clinical data
☐	☐	Dual use research of concern

Methods

n/a	Involved in the study	
☒	☐	ChIP-seq
☒	☐	Flow cytometry
☒	☐	MRI-based neuroimaging

Antibodies

Antibodies used	anti-SDHA monoclonal antibody (Cell Signaling Technology, Danvers, MA, USA; catalog number: 11998; 1:1000), anti-ACADV polyclonal antibody (Novus Biologicals, Centennial, CO, USA; catalog number: NBP2-15238; 1:500), anti-GAPDH monoclonal antibody (Proteintech Group Inc, Rosemont, IL, USA; catalog number: 60004-1-Ig; 1:50000), rabbit HRP-linked IgG (Cell Signaling Technology, Danvers, MA, USA; catalog number: 7074; 1:1000).
Validation	All antibodies were obtained from commercial sources and the validation data is available on the vendor websites--sufficient information is provided to easily locate this information.

Eukaryotic cell lines

Policy information about cell lines and Sex and Gender in Research

Cell line source(s)	Human oral squamous cell carcinoma (OSCC) cell lines derived from the gingiva (Ca9-22), tongue (SAS), and a site of lymph node metastasis (HSC-3) were obtained from the Human Science Research Resources Bank (Osaka, Japan). The immortalized normal human epidermal keratinocyte cell line (HaCaT) was obtained from Cell Lines Service (Eppelheim, Germany).
Authentication	All cell lines were obtained from commercial sources which each authenticated cell lines before shipping.
Mycoplasma contamination	Cell lines tested negative for Mycoplasma.
Commonly misidentified lines (See ICLAC register)	Not applicable.

Clinical data

Policy information about clinical studies

All manuscripts should comply with the ICMJE guidelines for publication of clinical research and a completed CONSORT checklist must be included with all submissions.

Clinical trial registration	NCT02415881
Study protocol	The study protocol (Stanford IRB-35064) is available from Dr. Rosenthal or the corresponding author upon reasonable request.
Data collection	Enrolled patients underwent surgery during which tissue samples were collected. Samples underwent histopathology assessment (hematoxylin and eosin (H&E) slides) and immunohistochemistry.
Outcomes	Not applicable.

Dual use research of concern

Policy information about dual use research of concern

Hazards

Could the accidental, deliberate or reckless misuse of agents or technologies generated in the work, or the application of information presented in the manuscript, pose a threat to:

No	Yes	
☒	☐	Public health
☒	☐	National security
☒	☐	Crops and/or livestock
☒	☐	Ecosystems
☒	☐	Any other significant area

Experiments of concern

Does the work involve any of these experiments of concern:

No	Yes	
☒	☐	Demonstrate how to render a vaccine ineffective
☒	☐	Confer resistance to therapeutically useful antibiotics or antiviral agents
☒	☐	Enhance the virulence of a pathogen or render a nonpathogen virulent
☒	☐	Increase transmissibility of a pathogen
☒	☐	Alter the host range of a pathogen
☒	☐	Enable evasion of diagnostic/detection modalities
☒	☐	Enable the weaponization of a biological agent or toxin
☒	☐	Any other potentially harmful combination of experiments and agents
